# The role of echocardiography in management of hypertrophic cardiomyopathy

**DOI:** 10.1007/s12574-019-00454-9

**Published:** 2019-12-19

**Authors:** Trine F. Haland, Thor Edvardsen

**Affiliations:** 1 Department of Cardiology, Oslo University Hospital, Rikshospitalet, Nydalen, PO Box 4950, 0424 Oslo, Norway; 2 University of Oslo, Oslo, Norway; 3 European Association of Cardiovascular Imaging, Sophia Antipolis, France

**Keywords:** Hypertrophic cardiomyopathy, Echocardiography, Systolic function, Diastolic function, Risk stratification

## Abstract

Hypertrophic cardiomyopathy (HCM) is the most common non-ischemic cardiomyopathy, characterized by increased left ventricular wall thickness. Echocardiographic studies are essential for establishing the diagnosis, evaluating the extent of disease, and risk stratification. Echocardiography is also recommended in regular screening of the genotype-positive relatives. Two-dimensional, M-mode, and Doppler echocardiography are standard modalities in HCM diagnosis. Newer echocardiographic techniques as tissue Doppler, strain, and three-dimensional echocardiography are now widely used and can reveal subtle changes in the HCM patients. Echocardiography has given us a better understanding of the disease. In this review, we briefly profile the echocardiographic management of HCM in a clinical perspective.

## Introduction

Hypertrophic cardiomyopathy (HCM) is the most common non-ischemic cardiomyopathy with a prevalence of 1:500 in the general population, based on the recognition of the phenotype [1]. HCM is caused by mutations in genes encoding proteins of the sarcomere protein in 50–70% of the cases [2–4]. HCM is defined by the presence of increased left ventricular (LV) wall thickness that is not solely explained by abnormal loading conditions and the phenotype also includes disorganized myocyte arrangement (disarray), fibrosis, small-vessel disease, and abnormalities of the mitral valve apparatus [5, 6]. The HCM is characterized by heterogeneous clinical expression and vary from asymptomatic or mildly symptoms to severe heart failure and sudden cardiac death. The penetrance of the mutation is not complete and genetic testing has created an important new group of patients, the genotype-positive relatives without signs and symptoms of HCM, but with the need of regularly clinical follow-up.

Echocardiography is an invaluable tool in diagnosis and follow-up of HCM patients, evaluating morphology, hemodynamic disturbances, LV function, and prognosis [7, 8]. Echocardiographic methodology has moved from linear measurements, via two-dimensional (2D) echocardiography with volume estimation, global, and regional deformation analysis to three-dimensional (3D) echocardiography [9]. This review briefly summarizes the most widely used echocardiographic techniques for diagnose and evaluation of adult HCM patients in a clinical perspective.

## Diagnosis

**Fig. 1:**
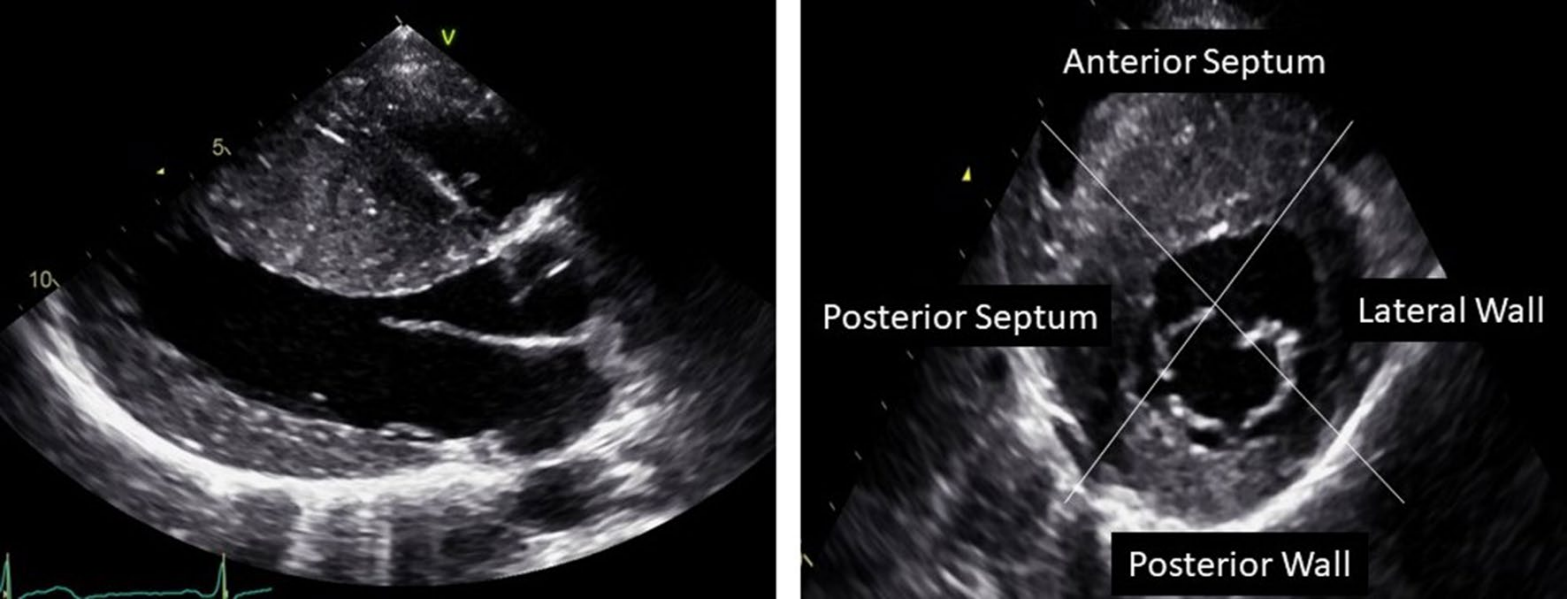
Parasternal long axis and short axis view of an HCM patient with distribution of hypertrophy especially in the septum with MWT of 30 mm

HCM diagnosis is linked to LV wall thickness ≥ 15 mm or maximal wall thickness (MWT) of ≥ 13 mm with the occurrence of a HCM-related mutation by any imaging modality (Table 1) [5]. Echocardiography is still the most important tool for diagnosis and clinical management of the HCM patients. Accurate assessment of MWT can be challenging and should be manually performed at end-diastole and preferably in short-axis views from the base to the apex of the LV (Fig. 1) to ensure that the MWT is measured at the mitral, mid-LV, and apical levels. M-mode can overestimate MWT by oblique cuts and should be avoided [5]. Three-dimensional echocardiography can help aiding the diagnosis and avoid over detection of MWT, including tendons and right ventricular moderator band.

**Table 1:** Diagnosis of HCM disease

HCM diagnosis
LV wall thickness ≥ 15 mm by any imaging modality
If HCM related mutation: LV wall thickness ≥ 13 mm

A characteristic feature of the pattern of hypertrophy in HCM is the asymmetric distribution that preferentially involves the intraventricular septum at the basal LV segments, with a septal to posterior free wall ratio > 1.3 [7]. The diagnosis of HCM patients with apical hypertrophy (10%) can be challenging and increased wall thickness may be ignored due to near-field artefacts. In cases of doubt, contrast echocardiography can be used to outline the endocardium [4, 5]. It can also be challenging to discriminate HCM patients with apical affection from the rarer non-compaction cardiomyopathy, because of increased trabeculation in both cardiomyopathies. Different deformation patterns by strain echocardiography can be used to discriminate between the two cardiomyopathies [10].

Nevertheless, genotype-positive relatives not fulfilling the strict HCM diagnosis have subtle changes in myocardial function compared with the normal population. This will be further discussed.

## Left ventricular outflow tract obstruction and mitral valve

It is of clinical importance to distinguish between the HCM with or without left ventricular outflow tract obstruction (LVOTO), because of different management strategies. Significant LVOTO is also related to worse prognosis and a predictor of heart failure and mortality in HCM patients [11]. LVOTO is dynamic and may vary with LV load and contractility. Approximately, one-third of the HCM patients are non-obstructive. One-third have a significant LVOTO defined as instantaneous peak Doppler pressure gradient ≥ 30 mmHg at rest (basal-obstructive) and one-third of the patients are labile-obstructive with significant gradient during provocation as Valsalva maneuver or exercise stress echocardiography. Pharmacological provocation is not recommended to detect labile-obstructive LVOTO and can be poorly tolerated (Table 2) [5]. Morphological features that contribute to LVOTO is systolic anterior motion (SAM) of the mitral valve [12]. The presence of SAM is best visualized by M-mode echocardiography characterized by mid-systolic notching of the aortic valve and contact of the anterior mitral valve with the septum. The severity of SAM is defined as mild if there is no mitral leaflet-septal contact with a minimum distance between the mitral valve and the ventricular septum of 10 mm. Severe SAM is defined as mitral leaflet-septal contact > 30% of systolic time [12]. The mechanism of SAM is widely discussed and can be caused by changes of the mitral valve with elongation of the anterior valve leaflet and drag forces with elevation and anterior movement of the mitral valve [13]. Because of failure in mitral valve leaflet coaptation, these findings are often followed by a laterally and posteriorly directed mitral regurgitation (MR). Transesophageal echocardiography (TEE) is recommended if presence of an anteriorly directed MR jet to exclude intrinsic mitral valve abnormality. For HCM patients with LVOTO related MR, invasive septal reduction can significantly reduce MR without mitral valve surgery. Other causes of LVOTO are small outflow tract dimension caused by hypertrophy, displacement, and hypertrophy of the papillary muscles [5, 14]. Isolated ventricular septal bulge (VSB) is fairly common in elderly and can cause LVOTO. The differentiation between VSB and septal HCM is difficult and not based on echocardiography alone [15]. Two-dimensional echocardiography is usually sufficient to evaluate LVOTO, but 3D echocardiography can give additional insights into the mechanism of SAM and geometry of the LVOT in selected patients. Some patients have limited image quality by transthoracic echocardiography, and TEE is recommended. This may detect the presence of sub-aortic membrane causing LVOTO, an important differential diagnosis to rule out.

**Table 2:** Diagnosis and management of left ventricular outflow tract obstruction in HCM patients

Left ventricular outflow tract obstruction
1. 1/3 are non-obstructive
2. 1/3 are obstructive (peak Doppler pressure gradient ≥ 30 mmHg at rest)
3. 1/3 are labile-obstructive with significant gradient during provocation or exercise
4. Pharmacological provocation is not recommended
5. Gradient of ≥ 50 mmHg is considered of hemodynamical importance
6. Myectomy or alcohol septal ablation (ASA) should be considered if the patients have moderate to severe symptoms and a gradient ≥ 50 mmHg

**Fig. 2:**
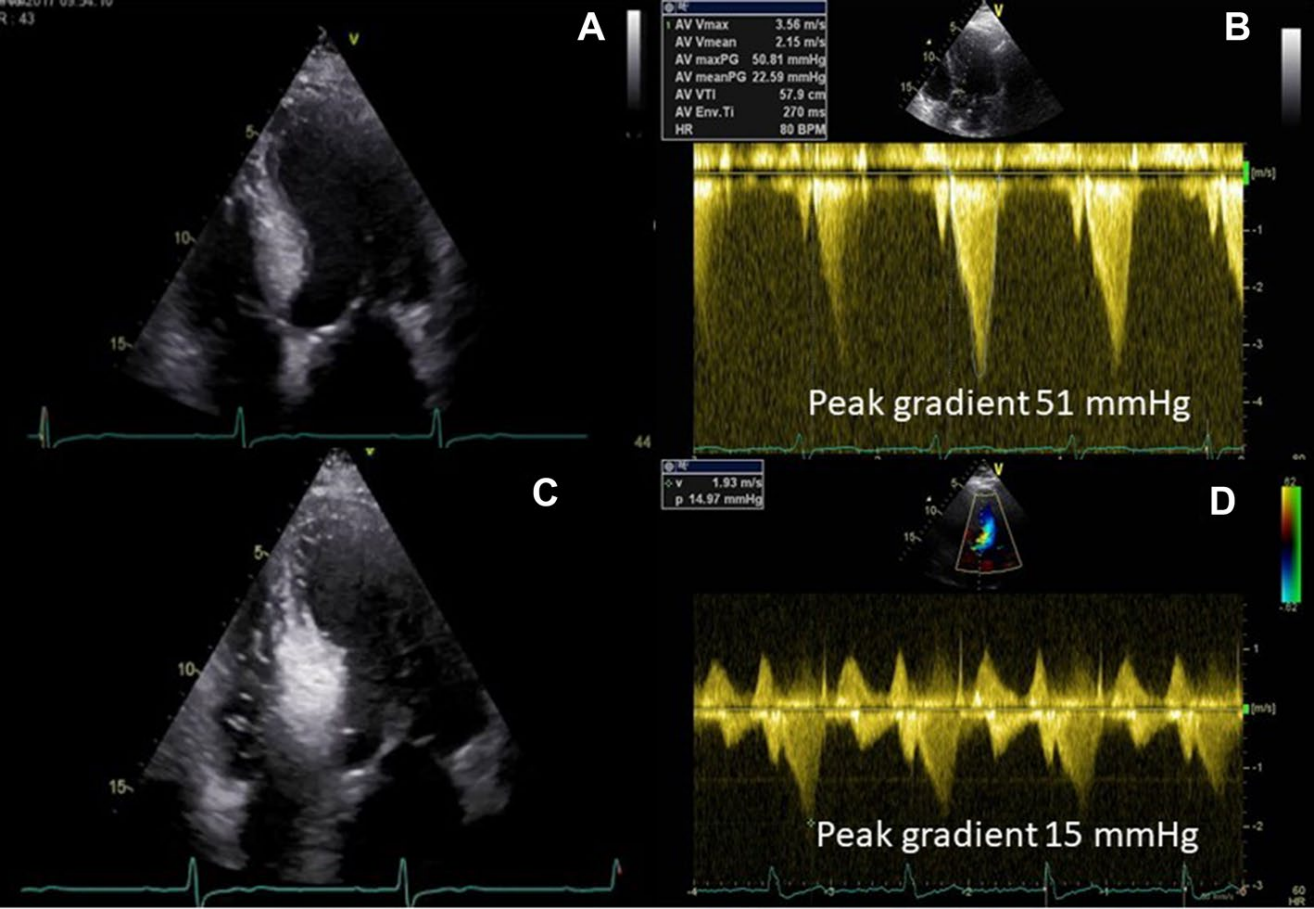
Patient with septal hypertrophy and MWT of 23 mm (**a**) with peak gradient of 51 mmHg at rest (**b**). Echocardiography with injection of contrast in septal branch of the coronary artery with supply of the basal part of the septum (**c**). Peak gradient of 15 mmHg after ASA (**d**)

A LVOTO gradient of ≥ 50 mmHg is considered of hemodynamical importance, and invasive treatment as myectomy or alcohol septal ablation (ASA) to reduce the gradient should be considered if the patients have moderate to severe symptoms (New York Heart Association function class III–IV, dizziness and syncope) despite medication (Table 2) [5]. TEE is used as intraoperative guidance during septal myectomy to reduce complications as ventricular septal defect and aortic regurgitation. TEE is also an important tool in the 11–20% of the patients undergoing concomitant mitral valve surgery [5]. Before ASA, myocardial contrast echocardiography is essential to find the septal branch to inject alcohol. During ASA, TEE is used to measure the fall in LVOT gradient and 2D echocardiography is a part of the clinical follow up evaluating the result before patients discharge (Fig. 2).

Elongation of the mitral leaflets can also be seen in genotype-positive relatives without LV hypertrophy and can be one of the hallmarks of HCM disease [16].

## Systolic function

The prognosis in cardiac diseases is closely related to LV systolic function [17]. LV ejection fraction (EF) is based on volume measurements and is the most widely used metric of LV systolic function despite its inherent weakness. EF is typically preserved in HCM patients, because of reduced LV volumes, despite significant impairment of longitudinal LV function measured with tissue Doppler velocity (TDI), and strain echocardiography [18]. EF is therefore not adequate to evaluate the indication for medical treatment and cardiac transplantation in HCM [5].

Measuring TDI has become standard in managing the HCM patients and systolic velocities should be performed at the basal infero septal and anterolateral walls routinely. Systolic myocardial velocity by TDI is reduced in HCM patients and has been shown to be attenuated even in non-hypertrophied segments and also in genotype-positive relatives [19]. It is important to be aware that angle dependency is an important limitation of TDI. Two-dimensional strain echocardiography bypasses this problem and can be measured through speckle-tracking echocardiography tracking acoustic markers (speckles) during the cardiac cycle, measuring the relative changes from end-diastolic to end-systolic dimensions. Speckle-tracking echocardiography is an excellent tool for assessing both regional and global myocardial functions. The technique allows evaluation of both longitudinal, circumferential and radial myocardial deformations [20, 21]. Despite normal EF, HCM patients have demonstrated worse global longitudinal strain (GLS) than healthy, but with increased circumferential strain and normal systolic torsion (Table 3) [10, 22]. The degree of hypertrophy is significantly correlated with worse GLS [23]. Despite reduced GLS, HCM patient often has a gradient with increasing longitudinal function by strain echocardiography from the LV base to the apex [10].

**Table 3:** Systolic function in HCM patients

LV systolic function
1. EF is typically preserved in HCM patients despite significant impairment of longitudinal systolic LV function
2. EF is therefore not adequate to evaluate medical treatment and cardiac transplantation
3. GLS by speckle-tracking echocardiography is an accurate measure of systolic function
4. Speckle-tracking echocardiography reveals subtle changes in systolic function in genotype-positive relatives

**Fig. 3:**
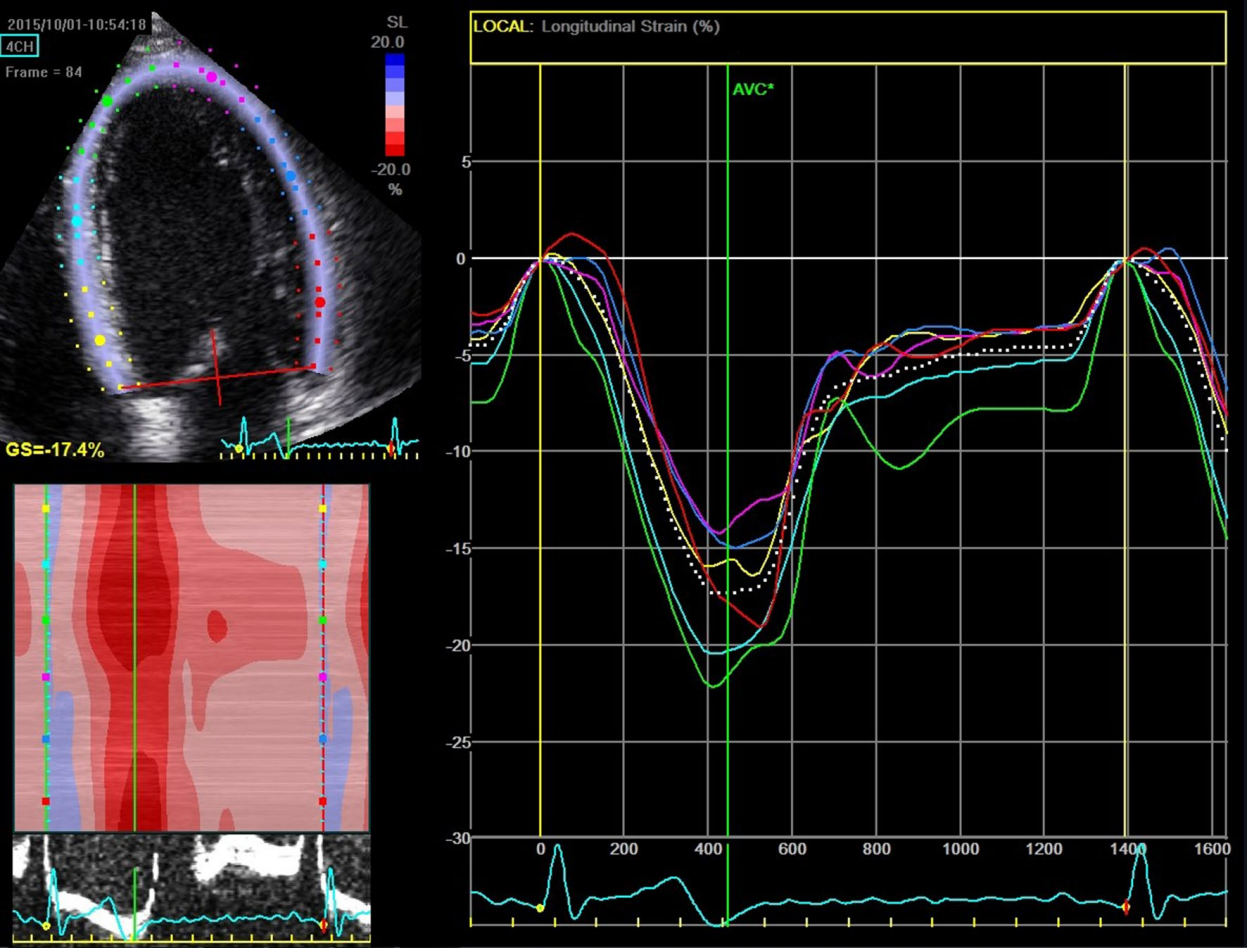
Longitudinal strain curves from apical four-chamber view in a 53-year-old genotype-positive (MYH7 mutation) relative with normal EF (63%). Average strain from four-chamber view was − 17% (dotted line) and GLS was − 18%, indicating reduced longitudinal function

Interestingly, studies have shown subtle changes in systolic function measured by TDI and strain analysis in genotype-positive relatives (Fig. 3) without increased wall thickness and normal EF [18, 24–26]. The hypertrophy can therefore be a compensatory mechanism for the induced abnormalities related to sarcomere mutations [18, 27–29].

## Diastolic function

Diastolic dysfunction is a major pathophysiological abnormality in HCM disease. The origin of diastolic dysfunction and increased LV filling pressure are multifactorial, including increased LV mass with reduction of chamber compliance, prolonged relaxation, ischemia, and myocardial fibrosis [12, 30]. HCM patients with restrictive filling pattern have higher risk of adverse outcome [31].

In the past, invasive measurements were required to determine the diastolic function by measuring the pulmonary capillary wedge pressure or LV end-diastolic pressure (LVEDP). Doppler echocardiography is a sensitive non-invasive parameter to evaluate diastolic function, but influenced by heart rate, age, and loading conditions. Doppler echocardiography, including trans-mitral flow velocities and TDI has allowed non-invasive estimation of filling pressure in other patients [32, 33]. However, it is important to be aware that non-invasive estimation using trans-mitral parameters as peak E wave (peak modal velocity in early diastole), *E*/*A* ratio (*E* velocity diastole divided by peak modal velocity in late diastole (*A*)), and deceleration time (time interval from peak *E* to zero velocity baseline) do not correlate well with LVEDP in HCM patients [12, 34]. $E/e^{\prime}$ ratio by TDI (using TDI-derived E velocity from the mitral annulus) provides more accurate estimate of LVEDP in HCM patients in some studies, but with modest correlation in others [34, 35]. A comprehensive approach is therefore recommended for the assessment of LV diastolic function, including multiple parameters as Doppler of mitral valve inflow, TDI at the mitral annulus, pulmonary vein flow velocities, left atrium (LA) size and volume, and peak velocity of tricuspidal regurgitation (TR) jet by continuous wave Doppler [5, 36]. According to ASE/EACVI guidelines, fulfilling more than 50% of the variables $E/e^{\prime}$ > 14, LA volume index > 34 mL/m^2^, pulmonary vein atrial reversal velocity (Ar-A duration ≥ 30 ms), and TR peak velocity of > 2.8 m/s are diagnostics for severe diastolic dysfunction in HCM patients (Table 4).

**Table 4:** Diastolic function in HCM patients

Diastolic dysfunction with elevated LVEDP is present in HCM patients if > 50% of the variables meet the cut-off values
1. $$ E/e^{\prime} $$ > 14
2. LA volume index > 34 mL/m^2^
3. Pulmonary vein atrial reversal velocity (Ar-A duration ≥ 30 ms)
TR peak velocity of > 2.8 m/s

Myocyte dysfunction and fibrosis are early abnormalities in HCM patients that can be seen in genotype-positive relatives without hypertrophy [27, 28]. Echocardiography has revealed lower early diastolic trans-mitral velocities and TDI in this population [24, 25]. It seems therefore that myocardial dysfunction occurs independent of hypertrophy.

## LA enlargement

LA is often enlarged in HCM patients, because of diastolic dysfunction and MR. It is important to recognize that use of linear dimensions may mispresent true LA size, because of asymmetric dilatation [7]. However, guidelines by European Society of Cardiology uses LA linear dimension ≥ 45 mm in recommendations for 6–12 monthly 48 h ambulatory ECG monitoring to detect atrial fibrillation, and in the risk calculator (Table 5) [5]. It has also been debated if HCM patients with increased LA should be treated with anti-coagulant independent of detection of atrial fibrillation, because of the high risk of developing atrial arrythmia. Compared with LA diameter, LA volume has a stronger association with adverse outcomes in cardiac patients [37]. Observational studies have showed that patients with atrial fibrillation and valvular disease with LA volume index ≥ 34 mL/m^2^ have higher risk of death, heart failure, atrial fibrillation, and ischemic stroke [38]. LA volume is highly feasible and reliable using LA volume (derived from biplane area length or method of disks) indexing to body surface. The limitation with this method is the geometric assumptions of the LA shape and 3D echocardiography measures the LA volume with more accuracy. Nevertheless, despite these advantages, there is lack of a consistent methodology and limited normative data of 3D measurements of LA [37].

**Table 5:** Risk stratification of sudden cardiac death in HCM patients

Risk stratification
HCM has an annual incidence of 1–2% sudden cardiac death. LV aneurysm increases risk of SCD and thromboembolic events
Risk calculator by European Society of Cardiology^a^
1. MWT
2. LA size
3. Maximal left outflow gradient
4. + age, family history of SCD, syncope, non-sustained ventricular tachycardia

^a^https://qxmd.com/calculate/calculator_303/hcm-risk-scd

Subtle changes with increased LA size have also been seen in the genotype- positive relatives compared to healthy [27].

## Risk stratification

Sudden cardiac death (SCD) is the most devastating complication of HCM, with an annual incidence of 1–2% [39]. The identification and treatment of patients with HCM who are at risk of SCD are important, but difficult [40]. Echocardiography is an important tool in risk stratification and HCM guidelines by the European Society of Cardiology includes MWT, LA size, and maximal left outflow gradient as a continuum in addition to age, family history of SCD, non-sustained ventricular tachycardia, and unexplained syncope to calculate the 5 years risk of SCD in HCM patients (Table 5) [5]. However, some HCM patients will come in an intermediate risk group using this current risk stratification and additional echocardiographic parameters may be used in decision making of cardioverter-defibrillator implantation as primary prevention. Potential arbitrators for malignant arrythmias and SCD are LV apical aneurysm, disarray, and fibrosis.

HCM patients with LV aneurysm are at risk for SCD and thromboembolic events (Table 5). Aneurism can easily be visualized by 2D echocardiography and the extent of the aneurism can be contained by 3D echocardiography. It is important to be aware that even small LV aneurysm can be a risk for thromboembolic events and the HCM patients should be evaluated for anticoagulation with warfarin [41].

**Fig. 4:**
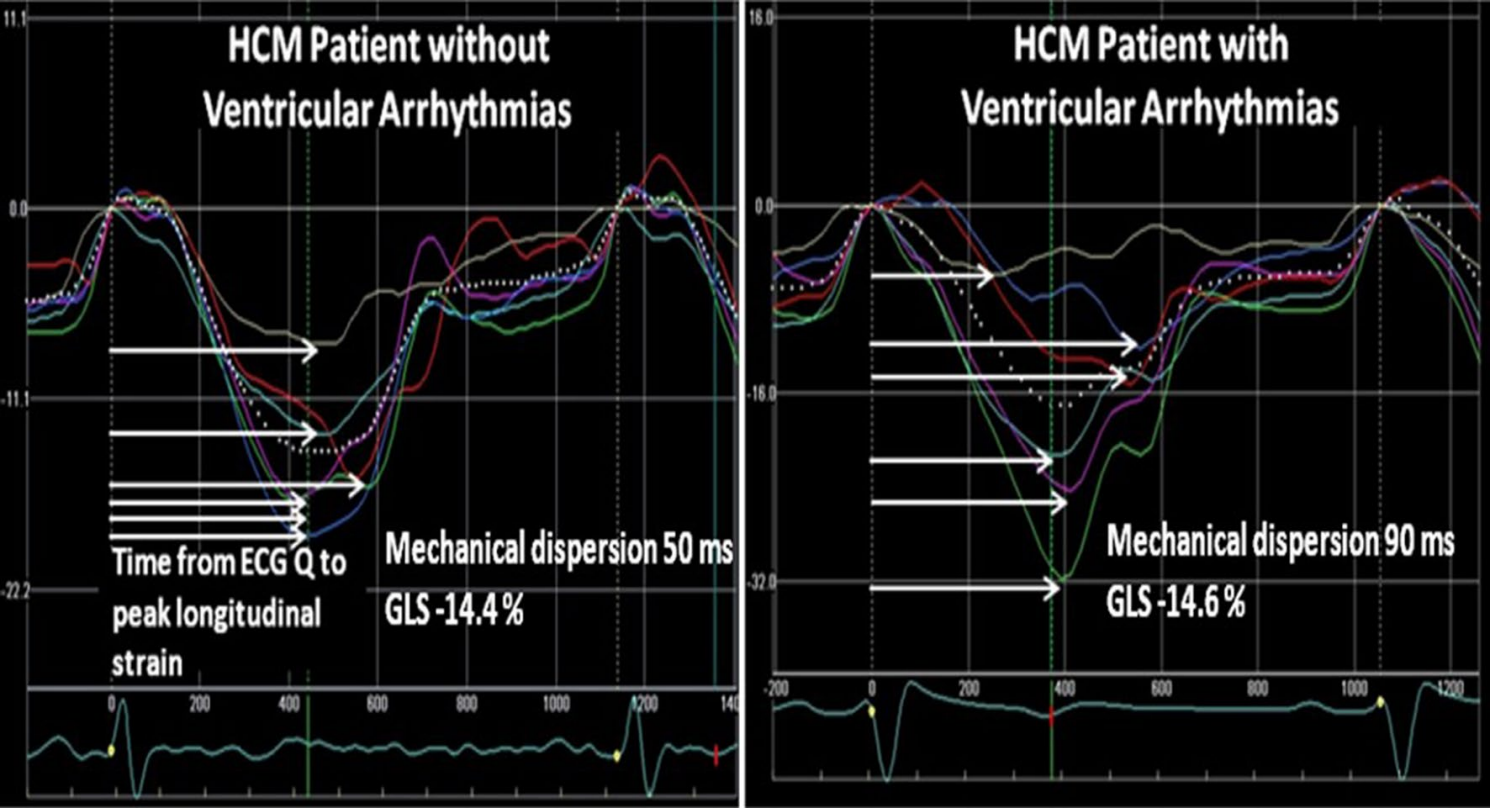
Mechanical dispersion by strain echocardiography in two patients with HCM. Horizontal white arrows indicate time to peak strain defined as the time from onset of Q/R to peak negative strain in each segment. Left panel displays longitudinal strain curves and mechanical dispersion in an HCM patient without ventricular arrhythmias. Left panel shows more pronounced mechanical dispersion in an HCM patient with ventricular arrhythmias

Extensive disarray with disorganized myocyte arrangement, microvascular ischemia, and fibrosis is also a potential mediator for SCD. Contrast-enhanced cardiovascular magnetic resonance (CMR) imaging can identify myocardial fibrosis. Though, CMR is time consuming and can be contraindicated, because of reduced kidney function. Heterogeneous contraction can be reflected by mechanical dispersion assessed by speckle-tracking strain echocardiography and may be related to electrical dispersion. Mechanical dispersion is defined as the standard deviation of time from onset Q/R wave on ECG to peak negative strain in 16 LV segments (Fig. 4) [42]. Mechanical dispersion has recently been demonstrated to relate to malignant ventricular arrhythmias in cardiomyopathies, and been demonstrated to relate to fibrosis by CMR in HCM patients [23, 43–45]. Mechanical dispersion may therefore be used as a marker of arrhythmias in addition to current risk scores, and when CMR is not available or contraindicated.

## Differential diagnosis

Differential diagnosis of HCM and other cardiac conditions with LV hypertrophy often arise, when MWT is in the modest range 13–15 mm with no history of HCM in the family.

### Hypertension

In patients with systemic hypertension, coexistence of HCM is often a consideration. However, the distribution of hypertrophy is regularly symmetric in patients with hypertension and MWT rarely exceeds 25 mm [46, 47]. Some studies has also showed that GLS is worse in HCM patients compared with hypertrophy related to hypertension [48].

### Athlete’s heart

HCM is an important cause of sudden cardiac death among young athletes [49]. Increased left wall thickness is a typical cardiovascular adaptation to athletic training and it can be difficult to distinguish between HCM patients and athletes. However, MWT in athletes is often not more than 13–16 mm with homogeneous distribution of hypertrophy, while HCM patients frequently have asymmetric distribution pattern. In addition, athletes often have dilated LV with end-diastolic diameter > 54 mm, as opposite to HCM patients with small LV cavity. Diastolic function is normal in athletes as contradictory to HCM patients, where diastolic dysfunction is one of the hallmarks of the disease [50].

### Cardiac amyloid

Amyloidosis present with increased MWT and can be difficult to differentiate from HCM patients. Increased myocardial echogenicity, symmetric hypertrophy, including the interatrial septum, right ventricle, increased thickness of the atrioventricular valves, and pericardial effusion are typical findings by echocardiography [12]. Longitudinal strain echocardiography has shown apical sparing with normal function in the apex in amyloidosis patients and can be used in differentiation of the two cardiomyopathies. This may indicate that relatively less amyloid deposition occurs in the apex than in the base [51].

### Fabry disease

The most common metabolic disorder in adults with hypertrophy is the X-linked Fabry disease. Patients with Fabry disease can have increased MWT caused by glycolipid deposition in ventricular muscle fibres. Concentrically increased wall thickness is the predominant pattern and the right ventricle may be affected. As in HCM patents, dilated LA, MR, and preserved systolic function by EF despite reduced LV function by strain echocardiography is seen. It is important to be aware that aorta at the level of sinus Valsalva and aorta ascendance can be dilated in Fabry disease, because of glycolipid deposition in the aortic wall [52]. Fabry disease is a difficult diagnose by echocardiography alone and often diagnosed by other clinical manifestation [12].

## Conclusion

Echocardiography is central to diagnose and in the clinical follow up of the HCM patients and the genotype-positive relatives. Comprehensive echocardiographic techniques are recommended to get an overview of the disease. Newer echocardiographic methods have revealed subtle changes in the genotype-positive relatives and additional research may give more information if these changes can tell us something about further prognosis in these relatives.
